# qPlus magnetic force microscopy in frequency-modulation mode with millihertz resolution

**DOI:** 10.3762/bjnano.3.18

**Published:** 2012-02-29

**Authors:** Maximilian Schneiderbauer, Daniel Wastl, Franz J Giessibl

**Affiliations:** 1 Institute of Experimental and Applied Physics, University of Regensburg, 93040 Regensburg, Germany

**Keywords:** hard disc, high-stiffness cantilever, magnetic force microscopy, qPlus

## Abstract

Magnetic force microscopy (MFM) allows one to image the domain structure of ferromagnetic samples by probing the dipole forces between a magnetic probe tip and a magnetic sample. The magnetic domain structure of the sample depends on the alignment of the individual atomic magnetic moments. It is desirable to be able to image both individual atoms and domain structures with a single probe. However, the force gradients of the interactions responsible for atomic contrast and those causing domain contrast are orders of magnitude apart, ranging from up to 100 Nm^−1^ for atomic interactions down to 0.0001 Nm^−1^ for magnetic dipole interactions. Here, we show that this gap can be bridged with a qPlus sensor, with a stiffness of 1800 Nm^−1^ (optimized for atomic interaction), which is sensitive enough to measure millihertz frequency contrast caused by magnetic dipole–dipole interactions. Thus we have succeeded in establishing a sensing technique that performs scanning tunneling microscopy, atomic force microscopy and MFM with a single probe.

## Introduction

Ferromagnetism is a collective phenomenon showing a parallel alignment of atomic magnetic dipole moments over macroscopic domains caused by a quantum-mechanical exchange interaction. Regions of aligned spins, called domains, are used, for example, to store bits of information on hard discs. Such ferromagnetic domains have much larger magnetic dipole moments, as many atoms contribute to the resulting moment.

**Figure 1:**
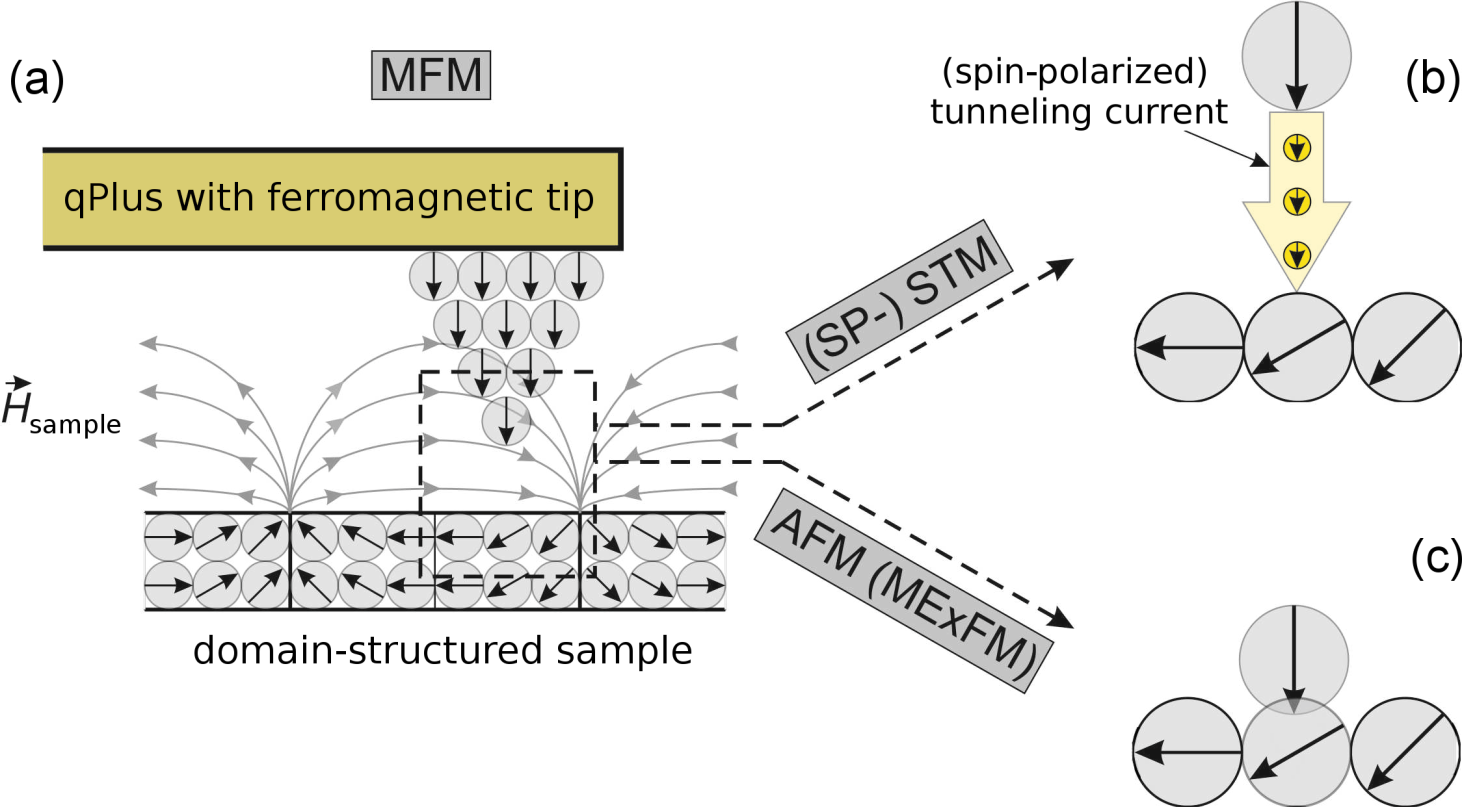
(a) MFM probes the force between the magnetic dipole moment of a probe tip and the magnetic stray field of a sample. With a qPlus sensor, the same probe can be used to perform (b) (SP-) STM and (c) AFM (MExFM) experiments.

To probe magnetic structures on the atomic as well as on the domain-size scale in real space, variations of Scanning Tunneling Microscopy (STM) [1] and Atomic Force Microscopy (AFM) [2] are used. To explore spin structures on conductive samples, the Spin Polarized-STM (SP-STM) [3–4] is a powerful tool. The SP-STM measures the spin-dependent conductivity between a spin-polarized tip and the spin-dependent local density of states of the sample (Figure 1b). STM is unable to probe insulating surfaces but AFM can be used: The antiferromagnetic surface structure of NiO (001) was imaged by Magnetic Exchange Force Microscopy (MExFM) [5]. In MExFM the magnetic exchange force between a tip atom with fixed spin orientation and a sample atom is measured (Figure 1c).

Imaging magnetic domains by Magnetic Force Microscopy (MFM) [6–7] is nowadays well-established. MFM images the magnetic-dipole interaction of a ferromagnetic tip and a domain-structured sample (Figure 1a). Typically, magnetically coated silicon cantilevers are used. These cantilevers are produced in large quantity by microfabrication techniques. Typical probe features are spring constants on the order of 10 Nm^−1^ and resonance frequencies of about 100 kHz. Another type of force sensor is made from a quartz (SiO_2_) tuning fork. The qPlus sensor [8] is based on a quartz tuning fork, in which one prong is attached to a carrier substrate. The large spring constant of the qPlus, *k* = 1800 Nm^−1^, allows one to overcome the snap-to-contact-problem in small-amplitude operation [9]. In this mode, the qPlus setup is customized for combined STM/AFM measurements with atomic resolution [10]. However, in standard MFM experiments, this large *k*, in combination with the resonance frequency *f*_0_ ≈ 31000 Hz, leads to very small frequency shifts (Equation 1).

Whereas MFM experiments employing quartz tuning forks, with both prongs oscillating, were previously conducted [11–12], the qPlus sensor has not yet proven its ability to detect weak long-range magnetic dipole interaction. In this article we show that the qPlus sensor is also capable of MFM experiments. We show imaging contrast of several millihertz in the large-amplitude regime, which is typically used for MFM. Therefore, we achieved a setup that is able to record a wide range of scanning-probe imaging signals; starting from domain-resolving MFM experiments, culminating in atomically resolved STM and AFM experiments (Figure 1).

## Results and Discussion

In frequency modulation AFM (FM-AFM) the measured frequency shift Δ*f* is proportional to an averaged force gradient 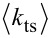 with *k*_ts_ = −*∂F_ts_*/*∂z*; *F*_ts_ is the force acting between tip and sample within one oscillation period; the *z*-direction is perpendicular to the sample surface. Within the gradient approximation, Δ*f* is given by:

[1]
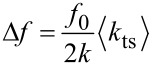


To determine the sensitivity of the experimental setup, and thus the minimum detectable averaged force gradient 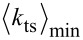, one has to calculate the frequency noise of the setup δ(Δ*f*). In FM-AFM setups δ(Δ*f*) is a sum of three uncorrelated noise sources [13–14]: Thermal noise

[2]
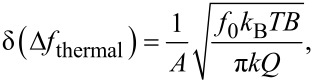


deflection-detector noise

[3]
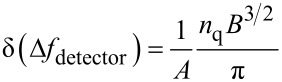


and oscillator noise

[4]
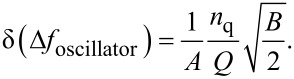


Here *A* is the cantilever amplitude, *f*_0_ the undisturbed resonance frequency of the cantilever, *k* the spring constant, *Q* the quality factor of the oscillation, *n*_q_ the deflection-noise density, *B* the bandwidth of the measurement, *k*_B_ the Boltzmann constant and *T* the temperature.

In each term, the frequency noise is inversely proportional to the oscillation amplitude *A* of the force sensor. Thus, we can reduce frequency noise by using large amplitudes and therefore minimize the 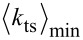. Moreover, one achieves the best signal-to-noise ratio by using an amplitude that is on the order of the decay length of the interaction being measured [15]. Here we take advantage of the large decay length of the magnetic dipole force, which is in the range of domain sizes, around 100 nm. Thus we chose oscillation amplitudes from 20 nm to 100 nm.

Typical values in our ambient qPlus setup are *f*_0_ ≈ 31000 Hz, *k* ≈ 1800 Nm^−1^, *Q* ≈ 2000, *B* ≈ 50 Hz, *n*_q_ ≈ 50 fm/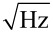 and *A* = 50 nm. This yields a frequency noise of δ(Δ*f*) ≈ 0.5 mHz. From Equation 1 we can now calculate the minimum detectable force gradient 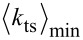 ≈ 5 × 10^−5^ Nm^−1^. In comparison, commercial silicon-cantilever setups with a standard MFM probe, *f*_0_ ≈ 75 kHz and *k* ≈ 3 Nm^−1^, are sensitive to force gradients down to 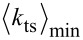 ≈ 5 × 10^−7^ Nm^−1^.

All experiments presented here were performed under ambient conditions. For vibration isolation the microscope is mounted on a mechanical double damping stage [16]. We used the Nanonis SPM [17] control electronics and the Multipass configuration to perform lift-mode experiments for MFM. The lift mode is a two-pass technique that enables a separation of topographic and, here, magnetic signals. In the first pass, a line is scanned in FM-AFM to obtain the topography of the surface. With the second pass, this previously acquired topographic trace is used to track the probe over the surface at an elevated tip–sample distance. Thus, the short-range van der Waals force is kept constant, and any force change is caused by long-range interactions, including the magnetostatic interaction. To minimize the long-range electrostatic interaction we compensated for the contact potential difference (CPD) in both paths. We determined the CPD by taking Kelvin parabolas over the sample surface; typical values are 250 mV. The Nanonis Multipass configuration also allows us to vary the scan speed on different paths. For the second path, in which the frequency shift is detected, we lowered the scan speed to half of the value used for topography imaging, thus reducing the detection bandwidth. As already mentioned, the oscillation amplitude should always be adapted to the interaction of interest. Thus, the lift-mode technique could be improved by programming a small amplitude for the topographic path and a large one for the magnetic path. In our current setup, the same amplitude is used for both paths. For FM detection we utilized the Nanonis OC4 and Nanosurf Saphyr, both of which are fully digital, allowing lowest noise operation. As a reference sample we used a 41 GB hard disc from MAXTOR with a bit density of approximately 2 Gbit/in^2^, resulting in a bit size of approximately (200 × 600) nm^2^.

Assuming a rigid tip magnetization in the *z*-direction, the magnetostatic force is a function of the magnetic moment of the tip and the gradient of the magnetic stray field of the surface [18]:

[5]
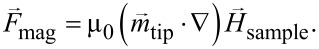


Here 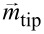 is the effective dipole moment of the probe and 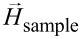 is the magnetic stray field of the sample. As 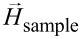 primarily varies in the *z*-direction, perpendicular to the sample surface, the main contribution of *F*_mag_ is given by the partial derivative in the *z*-direction. By using the same sample one can therefore vary the interaction strength by means of the magnetic moment of the tip and the lift-mode height.

**Figure 2:**
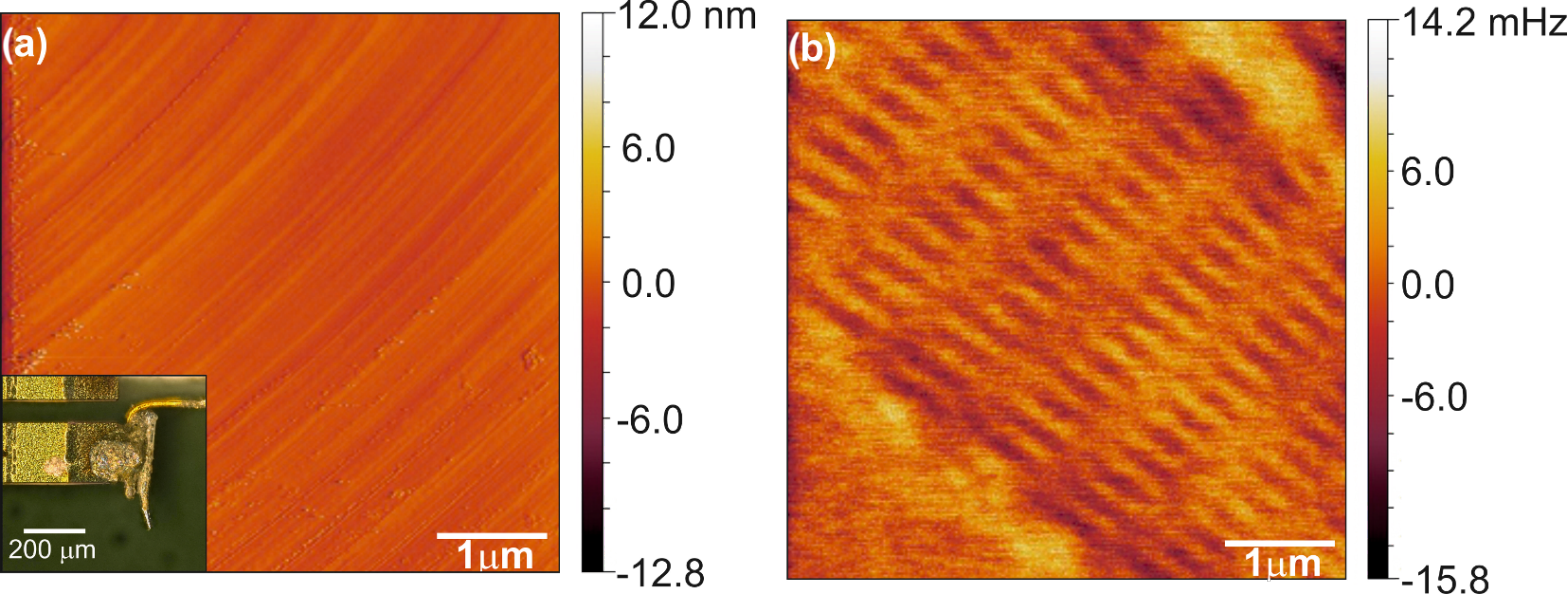
Lift Mode FM-MFM image using a qPlus sensor with an etched iron tip attached to it (see inset in a). Flattened raw data with imaging parameters *f*_0_ = 24097 Hz, *k* = 1250 Nm^−1^, *Q* = 1161, *A* = 20 nm and lift height 45 nm. (a) Topography and (b) lift-mode frequency shift.

In a first attempt we used an electrochemically etched bulk-iron tip (see inset in Figure 2a) and magnetized it for scanning by means of a strong permanent magnet. With this tip, and with an amplitude of 20 nm in both paths and a lift height of 45 nm, we imaged the bit structure of the hard-disc sample. The topographic image shows the typical surface texture of a hard disc (Figure 2a). The sizeable drift in both images is due to long measuring times, which were necessary in order to reduce the noise by reducing the bandwidth. In Figure 2b the flattened raw data of the frequency-shift channel gathered in lift-mode show an image contrast of ±5 mHz along the bit tracks. According to the resonance frequency *f*_0_ = 24097 Hz and spring constant *k* = 1250 Nm^−1^ of the sensor this contrast corresponds to a force gradient of ±520 μNm^−1^. The flat contrast in the upper-right and lower-left corner in Figure 2b is a marker region as we could measure another bit track beside it. The magnetic contrast in Figure 2b was also confirmed by scanning the same sample with a commercial silicon MFM cantilever setup (Nanosurf Flex AFM). Moreover we measured the expected bit density of ≈1.9 Gbit/in^2^ in Figure 2b.

As large magnetic moments of the probing tip can influence and even destroy the magnetic structure of the observed sample, a small magnetic moment is desirable. However, tips with a small magnetic moment reduce the interaction energy (Equation 5) and thus the signal strength, bringing the signal close to its noise floor. Here a trade-off has to be made between increased sensitivity due to decreased measurement bandwidth and large thermal drift at room temperature due to long acquisition times.

**Figure 3:**
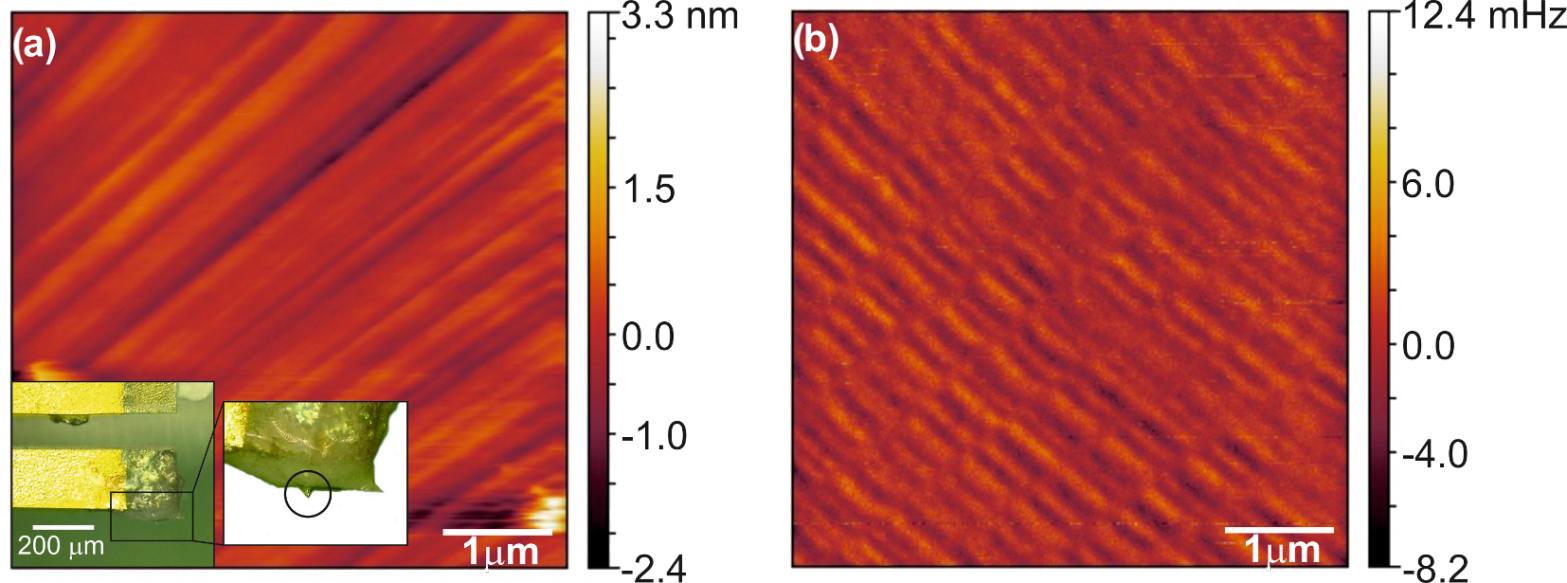
Lift Mode FM-MFM image employing a qPlus sensor with a commercial cobalt-coated MFM cantilever tip attached to it (see inset in a). Flattened raw data with imaging parameters *f*_0_ = 32517 Hz, *k* = 1800 Nm^−1^, *Q* = 1870, *A* = 25 nm and lift height 35 nm. (a) Topography and (b) lift-mode frequency shift.

To benchmark our setup, we reduced the magnetic moment of the tip by attaching a commercial MFM cantilever tip (NanoWorld Pointprobe MFMR, coated with approx. 40 nm cobalt alloy) onto a qPlus sensor. This has been done before in tuning-fork setups in room-temperature ultrahigh-vacuum systems [19] and low-temperature systems [12,20–21]. For this sensor setup, see inset in Figure 3a, we found an amplitude of 25 nm in both paths and a lift height of 35 nm to be a good choice. The first-pass topography data set shows the expected surface structure (Figure 3a). The scan speed again had to be set to relatively slow values, allowing for a small bandwidth, but leading to sizeable drift, as seen in both sets of Figure 3. The frequency-shift data set in the second (MFM) path was flattened by applying a simple parabolic fit and shows an image contrast of ±10 mHz (Figure 3b). Along the magnetic tracks, the frequency shift varies by ±2 mHz. Based on the properties of the sensor, *f*_0_ = 32517 Hz and *k* = 1800 Nm^−1^, this frequency shift corresponds to a force gradient of ±220 μNm^−1^.

## Conclusion

The key aim of this study was to find out if it is possible to observe the weak contrast caused by magnetic dipole interactions, with a qPlus force sensor that is optimized to detect the strong force gradients of chemical bonds. Chemical bonds show force gradients up to about 100 Nm^−1^, while we have shown here that a sensor with a stiffness of 1800 Nm^−1^ can resolve force gradients from magnetic dipole forces with a magnitude of only ±220 μNm^−1^. Therefore, we have clearly demonstrated that, although the relevant prefactor *f*_0_/*k* (Equation 1) is only about 20 Hz(N/m)^−1^ for the qPlus sensor versus 4000 Hz(N/m)^−1^ for standard Si cantilevers with *f*_0_ = 200 kHz and *k* = 50 Nm^−1^, it is perfectly feasible to perform magnetic force microscopy with qPlus sensors, even under ambient conditions.

State-of-the-art low-temperature magnetic force microscopy has been applied to measure the Barkhausen effect, yielding a frequency-shift contrast of 0.7 Hz for a cantilever with *f*_0_ = 195 kHz and *k* = 47 Nm^−1^ [22], which corresponds to a magnetic force gradient of 340 μNm^−1^. At low temperatures we expect that the noise in our MFM measurements will decrease dramatically due to an increase in *Q*, a decrease in *n*_q_ (Equation 2–Equation 4), and a decrease in thermal frequency drift, therefore we trust that qPlus sensors will become a competitive alternative to Si cantilevers for performing MFM under such conditions. The key benefit of employing the qPlus sensor in MFM, however, is that atomically resolved STM and AFM as well as MFM is possible without changing the probe.
